# Exploring the effects of competition and predation on the success of biological invasion through mathematical modeling

**DOI:** 10.1038/s41598-024-53344-1

**Published:** 2024-02-22

**Authors:** Viviana Rivera-Estay, Fernando Córdova-Lepe, Felipe N. Moreno-Gómez, Hugo Benitez, Rodrigo Gutiérrez

**Affiliations:** 1 Doctorado en Modelamiento Matemático Aplicado, Facultad de Ciencias Básicas, Universidad Católica del Maule, 3466706 Talca, Chile; 2 Departamento de Matemática, Física y Estadística, Facultad de Ciencias Básicas, Universidad Católica del Maule, 3466706 Talca, Chile; 3 Departamento de Biología y Química, Facultad de Ciencias Básicas, Universidad Católica del Maule, 3466706 Talca, Chile; 4 Laboratorio de Ecología y Morfometría Evolutiva, Centro de Investigación de Estudios Avanzados del Maule, Instituto Milenio Biodiversidad de Ecosistemas Antárticos y Subantárticos (BASE), Universidad Católica del Maule, 3466706 Talca, Chile; 5 Centro de Investigación en Recursos Naturales y Sustentabilidad (CIRENYS), Universidad Bernardo O’Higgins, Avenida Viel 1497, 8370993 Santiago, Chile

**Keywords:** Invasive species, Population dynamics, Ecological modelling, Applied mathematics

## Abstract

Biological invasions are a major cause of species extinction and biodiversity loss. Exotic predators are the type of introduced species that have the greatest negative impact, causing the extinction of hundreds of native species. Despite this, they continue to be intentionally introduced by humans. Understanding the causes that determine the success of these invasions is a challenge within the field of invasion biology. Mathematical models play a crucial role in understanding and predicting the behavior of exotic species in different ecosystems. This study examines the effect of predation and competition on the invasion success of an exotic generalist predator in a native predator-prey system. Considering that the exotic predator both consumes the native prey and competes with the native predator, it is necessary to study the interplay between predation and competition, as one of these interspecific interactions may either counteract or contribute to the impact of the other on the success of a biological invasion. Through a mathematical model, represented by a system of ordinary differential equations, it is possible to describe four different scenarios upon the arrival of the exotic predator in a native predator-prey system. The conditions for each of these scenarios are described analytically and numerically. The numerical simulations are performed considering the American mink (*Mustela vison*), an invasive generalist predator. The results highlight the importance of considering the interplay between interspecific interactions for understanding biological invasion success.

## Introduction

Biological invasions are one of the major drivers of current species extinction and biodiversity loss^1–4^. Understanding the causes that determine the success of these invasions is a challenge within the field of invasion biology, as it allows the improvement of early detection, prevention and management programmes^5,6^. Extensive research efforts have focused on introduction history, species traits, and ecological and evolutionary processes^7,8^. Based on this, it is possible to define three key factors that determine the success of a biological invasion: invasiveness of the exotic species, invasibility of the site, and propagule pressure^9–11^.

The invasiveness of the exotic species corresponds to its ability to become established, while the invasibility of the site corresponds to the abiotic and biotic components of the receiving ecosystem to be invaded, which include the characteristics of the native species^5,10,12^. Relative differences in the demographic factors exhibited by both exotic and native species play a crucial role in interspecific interactions and consequently in the success of the invasion^13–19^. Regarding the propagule pressure, it corresponds to the number of introduction events and the number of individuals introduced in each of these events^9,11,20^. The initial propagule size (*i.e.*, number of individuals) necessary for establishment depends on the invasibility of the site^21,22^.

The arrival of an exotic species in a native predator-prey system can have a significant impact on ecosystems and consequently disrupt the balance of biodiversity^23–25^. The new interspecific interactions that occur depend on the role that the exotic species assumes. In some cases, exotic species may emerge as competitors, potentially challenging one or both of the native species, either directly or indirectly through resource use. Although competition by interference is indirect competition for the resource, it can involve direct negative interactions arising from territoriality, overgrowth, predation or chemical competition^26,27^. The exotic invasive species that displace native fauna and flora appear to do so via superiority in interference competition^28–30^. Alternatively, exotic species may assume the role of potential predators for which native prey lacks defense or escape mechanisms^31^. Predator behavior and its effects on prey populations can be described by the functional response^32^. An exotic predator with a Holling Type-I functional response exhibits a linear increase in feeding rate as it encounters more prey, simplifying the comparison between native and exotic predator rates. For example, if the exotic predator consumes more prey than the native predator, the native prey population may decrease, and consequently, native predator population may also decline^33–35^.

An exotic predator in a native predator-prey system may simultaneously consume the native prey and indirectly compete with the native predator for resources^36,37^. In this context, invasion success depends on predation pressure and the intensity of competition imposed by the exotic. These interspecific interactions may either counteract or contribute to the effects of the other on the success of a biological invasion^38,39^. Therefore, it is important to consider the interplay between predation and competitive interactions in the establishment of the exotic predator.

Empirical studies of biological invasions have been used to understand the factors influencing the establishment of exotic species^40,41^. However, these studies have faced challenges in collecting information, such as geographical limitations that limit their scope in different ecosystems and long observation periods to capture long-term changes in invaded ecosystems^42^. Mathematical models provide a powerful tool for ecologists and conservationists to analyze and interpret complex ecological processes^43,44^. These models play a crucial role in understanding and predicting the behavior of exotic species in different ecosystems^45–48^.Through their study and analysis, it is possible to explore hypothetical scenarios, assess the long-term consequences of invasive species and evaluate the effectiveness of different control measures. For instance, Gutierrez and Teem proposed a novel method to induce the extinction of an exotic fish population using a genetic approach^49^. Mougi showed that adaptive trait dynamics can lead to cyclic coexistence between native and exotic species, even when their ecological traits are very similar and interspecific competition is strong^27^. Jones et al. have contributed to the conservation of red squirrels across the UK by studying the dynamics of competitive and epidemiological interactions in the red-grey-squirrelpox system^47^. Inoue provides a method for quantitatively assess the impact of bycatch on native populations and to determine the conditions under which only invasive species are removed^50^.

This study uses a mathematical model to investigate the effects of competition and predation, as well as their interaction, on the success of a biological invasion. Notably, the analysis focuses on the invasion success during the establishment stage of the biological invasion process^51^. The model is represented by a system of ordinary differential equations and describes the population dynamics of a native predator-prey system, in which an exotic predator consumes the native prey and competes with the native predator. Predation rates and competition coefficients of both predators are assumed to be proportional, simplifying the comparison of the predation pressure and competition intensity of the exotic predator in relation to the native predator. General analytical and numerical results of the model allow the determination of the conditions necessary for invasion success, highlighting the importance of considering the interplay between interspecific interactions. Furthermore, to exemplify the modeling approach, numerical simulations are performed considering the American mink (*Mustela vison*). This invasive species imposes risks to native wildlife, is a potential generalist predator responsible for declines in native preys including birds^52^ and mammals^53^. Moreover, it had serious negative impacts on native competitors such as *Lutra lutra* and *Lontra provocax* in Europe and South America, respectively^36,37^.

## The model

We formulated a mathematical model to describe the dynamics of native and exotic populations. First, the model considers only native predator and prey species to establish baseline conditions. Then the exotic predator is introduced and the three species are considered together by modifying this two-species model, which is termed native predator-prey system. In the two-species native model, $x = x(t)$ and $y = y(t)$ correspond to the population densities of the prey and predator species as a function of time *t*, respectively. The two-species native model is formulated by deterministic coupled differential equations based on the classical Lotka-Volterra predation model^54,55^:

1 $$\begin{aligned} \left\{ \begin{aligned} \frac{dx}{dt}&= f(x,y):= rx\left( 1-\frac{x}{K}\right) -dxy \\ \frac{dy}{dt}&= g(x,y):=pdxy-qy, \end{aligned} \right. \end{aligned}$$

where prey population growth is described by a simple logistic equation and is reduced by encounters with predators. Predator population growth depends on prey consumption and decreases exponentially in the absence of prey. The standard ecological parameters *r*, *K*, *d*, *p* and *q* are defined in Table 1. This model was studied in^56^. The equilibrium points and their stability conditions are shown in Table 2. It is important to note that, when the mortality rate *q* exceeds or equals the theoretical maximum birth rate of the prey population (when the prey population is *K*), the predator becomes extinct.

**Table 1:** Ecological meaning of parameters in the model (1) and model (2).

Parameter	Ecological meaning	Unit
*r*	Intrinsic growth rate of the native prey	$t^{-1}$
*s*	Intrinsic growth rate of the exotic predator	$t^{-1}$
*K*	Carrying capacity of native prey	*indiv*.
*d*	Predation rate of the native predator	$(indiv.t)^{-1}$
*n*	Enlarger of carrying capacity	*indiv*.*t*
*c*	Carrying capacity minimum of exotic predator guaranteed by the alternative food	*indiv*.
*b*	Competition coefficient	$(indiv.t)^{-1}$
*p*	Conversion coefficient: food intake to new native predator	unitless
*q*	Mortality rate of the native predator	$t^{-1}$
$\alpha$	Constant of proportionality between predation rates	unitless
$\beta$	Constant of proportionality between competition coefficients	unitless

**Table 2:** Existence and stability conditions of the equilibria of the model (1). When $pdK=q$ the equilibria $e_{x}$ and $e_{xy}$ are equals.

Equilibria	Existence condition	Stability condition
$e_0=(0,0)$	Always	Always unstable
$e_x=\left( K,0\right)$	Always	$pdK<q$
$e_{xy}=\left( \frac{q}{dp},\frac{r(pdK-q)}{d^{2}Kp}\right)$	$pdK>q$	Always

**Figure 1:**
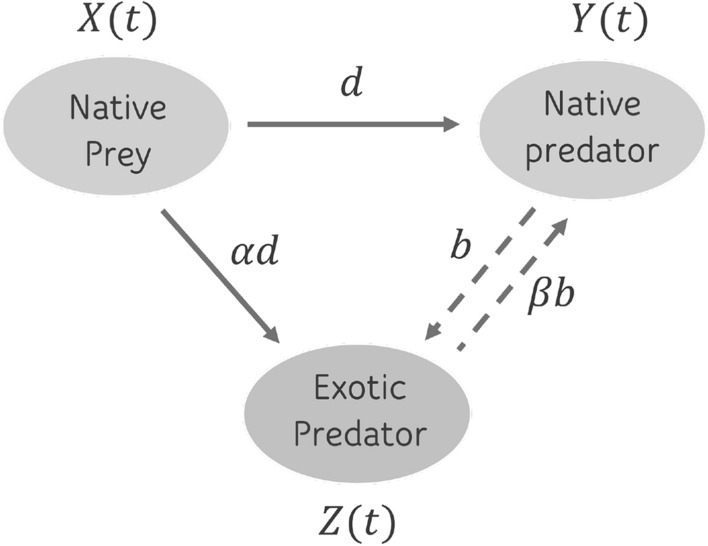
Interactions diagram between native and exotic populations. Solid arrows denote the decrease in prey population due to predation. Dashed arrows indicate the population decrease due to competition on both native and exotic predator populations.

In the formulation of three species model (native prey and predator, plus exotic predator, as shown in Fig. 1), the following assumptions are made:

(i) The biological system consists of three species: the native prey, the native predator, and the exotic predator. Their densities over time are denoted by $X=X(t)$, $Y=Y(t)$ and $Z=Z(t)$, respectively.
(ii) Native prey follows a logistic growth with carrying capacity *K* and intrinsic growth rate *r*. Its growth is reduced by encounters with native and exotic predators.
(iii) The native predator has a specialist feeding strategy, which means that in the absence of prey population, the native predator population declines exponentially with a per capita mortality rate *q*.
(iv) The exotic predator has a generalist feeding strategy. Its carrying capacity depends on the size of the prey population *X* and other available food *c*. Therefore, even in the absence of the prey, the exotic predator population follows a logistic growth with carrying capacity *c* and at an intrinsic growth rate *s*.
(v) The native and exotic predators have constant consumption rates. Then, the per capita consumption rate of both predators on the native prey is represented by a linear functional response^32^.
(vi) It is assumed that both predation rates are proportional, so if $\alpha$ is the positive constant of proportionality, then *d* and $\alpha d$ are per capita consumption rates of the native predator and exotic predator, respectively. Importantly, if $\alpha >1$, the exotic predator consumes more prey relative to the native predator. Conversely, if $\alpha <1$, the exotic predator consumes less prey relative to the native predator. This makes it easier to compare the effect of the two predators on the prey population. Furthermore, the efficiency with which native prey is consumed leads to the production of native and exotic predators, parameters denoted by *p* and *n*, respectively.
(vii) Competition coefficients are proportional, such that if $\beta$ is a positive constant of proportionality, then *b* and $\beta b$ are the mortality rates by competition of the exotic predator and native predator, respectively. Note that, if $\beta >1$, the exotic predator is a better competitor relative to the native predator. Conversely, if $\beta <1$, the exotic predator is a worse competitor relative to the native predator. This allows a comparison of the effect of competition between both predators.

Given the above assumptions, the mathematical model is described by the following system of equations:

2 $$\begin{aligned} \left\{ \begin{aligned} \frac{dX}{dt}&= f(X,Y)-\alpha dXZ \\ \frac{dY}{dt}&= g(X,Y)-\beta bYZ\\ \frac{dZ}{dt}&= sZ\left\{ 1-Z/\kappa (X)\right\} - bZY,\,\,\,\,\text{ with }\,\, \kappa (X):=n\alpha dX+c, \end{aligned} \right. \end{aligned}$$

where *r*, *K*, *s*, *n*, *c*, *b*, *d*, $\alpha$, $\beta$, *p*, *q* are positive parameters (see Table 1). Observe that if $Z = 0$ for any $t\ge 0$, the system (2) reduces to (1). In addition, in marginal terms, *i.e.*, in the increase from *Z* to $Z+1$, the loss in birth and the increase in mortality due to intraspecific competition is $s/\{n\alpha d X+c\}$, this is decreasing with respect to *X* from a maximum *s*/*c*. In order to make a proportional comparison between the predation rates of the native and exotic predators, a direct qualitative analysis of the system (2) is conducted, explicitly maintaining the parameter *d*.

## Results

### Native predator-prey system

**Figure 2:**
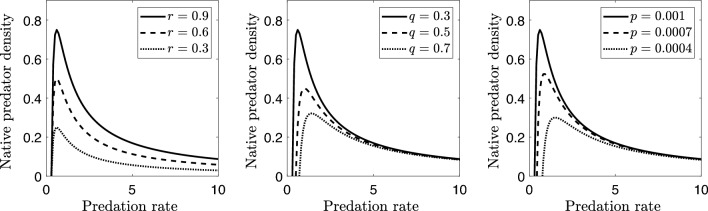
The variation of the native predator density $y_{s}$ in relation to the predation rate *d* for different values of *r*, *q* and *p*. As parameter *d* increases, the native predator density first increases until it reaches a maximum and then decreases.

In the native predator-prey system (1) the coordinates of the positive equilibrium $e_{xy}=\left( \frac{q}{dp},\frac{r(pdK-q)}{d^{2}Kp}\right)$ depends on the parameters *r*, *K*, *p*, *q* and *d*, whose ecological meanings are presented in Table 2. The predation rate *d* has a considerable effect on the dynamics of the model in contrast to other parameters. Predator density has a positive correspondence with *r*, *K*, and *q*, while it shows a negative correspondence with *p*. However, the population predator may increase or decrease as *d* increases (see Fig. 2).

#### Proposition 1

In native predator-prey system (1) the equilibrium $y_s$ as a function of *d*: (a) increases rapidly if $0<d < d^{*}$, (b) decreases slowly if $d>d^{*}$, or (c) reaches its peak if $d=d^{*}$, where $d^{*}=2q/(Kp)$.

#### Proof Proposition 1

The predator population at equilibrium, as a function of *d*, is given by $y_{s}=r \left( d-d^{*}/2 \right) /d^{2}$. Considering that its derivative is $\partial y_s/\partial d=r(d^{*}-d)/d^{3}$, assertions (a), (b), and (c) are clear analyzing its signs.

#### Remark 1

It can be observed that the predator equation with $d=d^{*}$ takes the form $dy/dt=qy\{x/(K/2)-1\}$ from which it can be concluded that when the prey population reaches its maximum natural growth at *K*/2, the predator population also reaches its peak value. This finding suggests an optimized logistic balance between the two species.

### Including an exotic predator into the native predator-prey system

**Figure 3:**
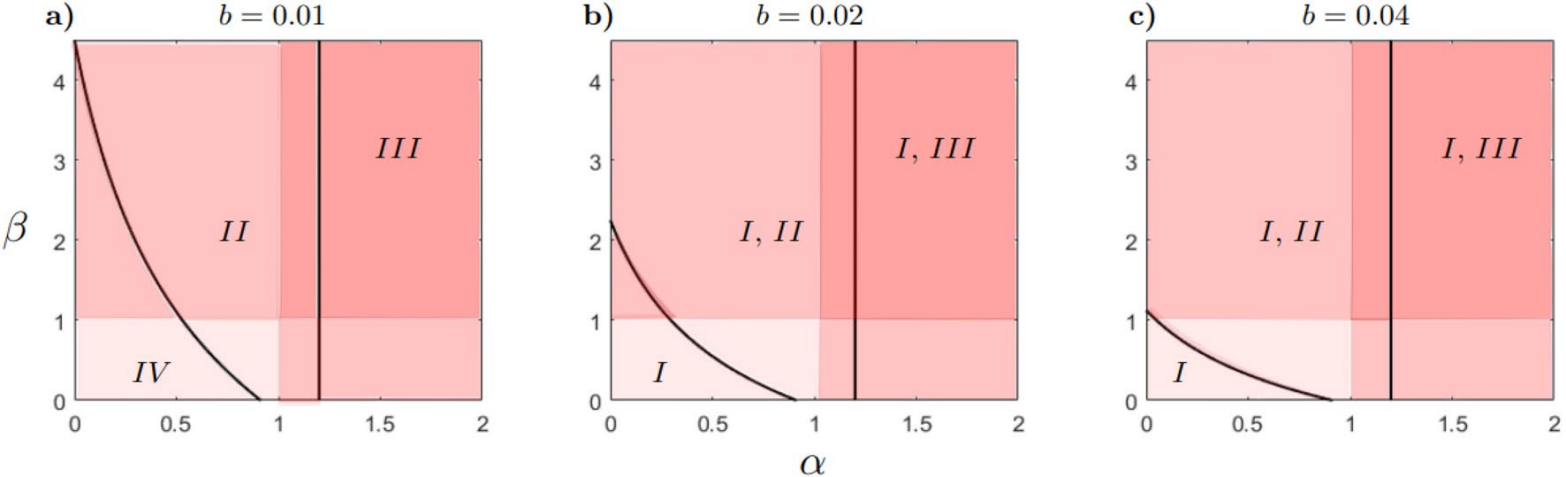
Bifurcation diagram in the $(\alpha ,\beta )$-plane, for different values of parameter *b* and fixed $r=1.2$, $s=0.2$, $K=100$, $c=10$, $n=2$, $p=0.05$, $q=0.05$ and $d=0.1$. The initial point $\left( \alpha _{0},\beta _{0},X_{0},Y_{0},Z_{0}\right)$ for the numeric continuation of the bifurcation curve are: panel (**a**) (0.505, 1.086, 40.625, 0.0045, 14.1), panel (**b**) (0.505, 0.543, 40.6421, 0, 14.1049) and panel (**c**) (0.505, 0.271, 40.6421, 0, 14.1049). The colors represent the intensity of predation and competition by the exotic predator compared to the native predator. The light color indicates low predation and competition. The medium color suggests lower predation and high competition (or vice versa). The dark color suggests high predation and competition.

**Figure 4:**
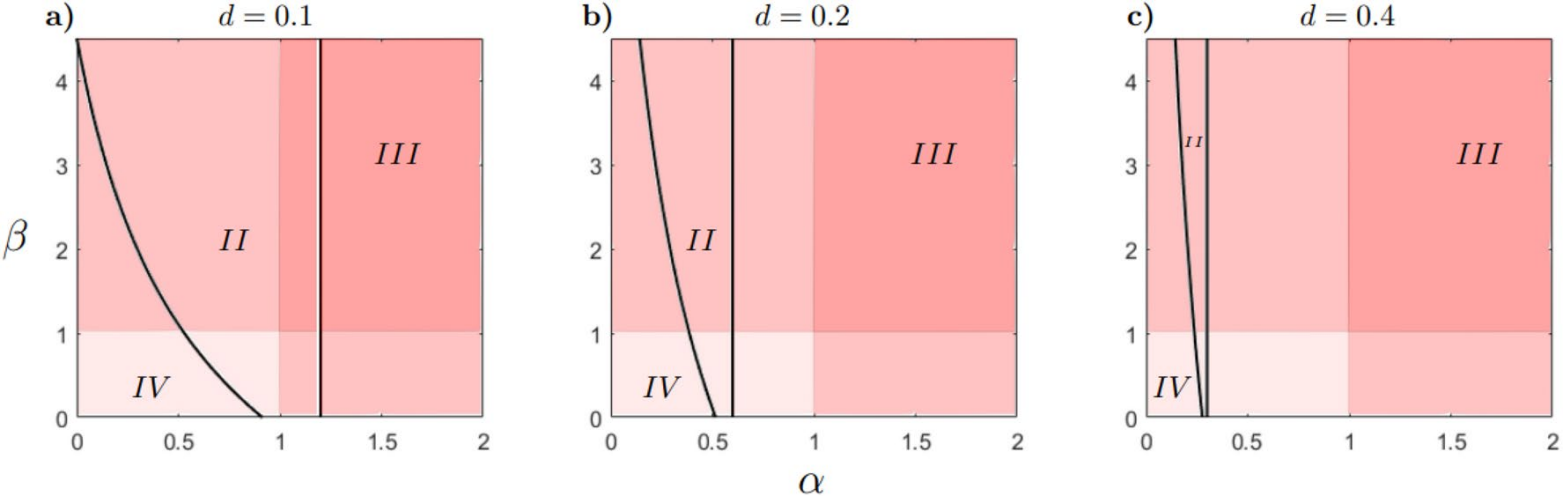
Bifurcation diagram in the $(\alpha ,\beta )$-plane, for different values of parameter *d* and fixed $r=1.2$, $s=0.2$, $K=100$, $c=10$, $n=2$, $p=0.05$, $q=0.05$ and $b=0.01$. The initial point $\left( \alpha _{0},\beta _{0},X_{0},Y_{0},Z_{0}\right)$ for the numeric continuation of the bifurcation curve are: panel (**a**) (0.505, 1.086, 40.625, 0.0045, 14.1), panel (**b**) (0.505, 0.076, 8.922, 4.874, 7.768) and panel (**c**) (0.242, 0.869, 7.471, 0.0067, 11.442). The colors represent the intensity of predation and competition by the exotic predator compared to the native predator. The light color indicates low predation and competition. The medium color suggests lower predation and high competition (or vice versa). The dark color suggests high predation and competition.

**Figure 5:**
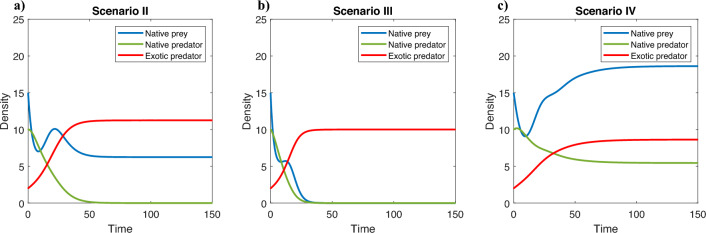
Different establishment scenarios for the exotic predator. From left to right values of $\alpha$ and $\beta$ in region *II*, *III* and *IV*. Fixed $r=1.2$, $s=0.2$, $K=100$, $c=10$, $n=2$, $p=0.05$, $q=0.05$, $b=0.01$ and $d=0.1$. In all panels, the initial conditions for native prey, native predator and exotic predator populations are $X_{0}=15$, $Y_{0}=10$ and $Z_{0}=2$, respectively.

The model (2) has a maximum of seven equilibrium points of ecological interest. The existence and stability conditions for each equilibrium are provided in Table 3 (see Stability conditions in Supplementary Material for more details). Taking these conditions into account, the parameter space $(\alpha , \beta )$ is partitioned into different regions as shown in Figs. 3 and 4. Each region represents a different scenario in which an exotic propagule enters a native predator-prey system in equilibrium. In scenario *I*, the exotic population cannot grow, therefore the native system remains in equilibrium, that is, $E_{XY}$ is asymptotically stable. In scenario *II*, the exotic population grows and the native predator declines to extinction. Coexistence of the exotic predator and native prey is possible, thus $E_{XZ}$ is asymptotically stable (Fig. 5a). In scenario *III* the exotic population grows and the native prey declines to extinction. Consequently, the native predator also declines to extinction as well, that is, $E_{Z}$ is asymptotically stable (Fig. 5b). In scenario *IV* the exotic population grows and the system reaches a new equilibrium in which the three species show positive densities, that is, $E_{s}$ is asymptotically stable (Fig. 5c). Thus, *II* and *III* correspond to scenarios of invasion success, as they involve the extinction of one and both native species, respectively. In turn, *IV* could correspond to a successful invasion scenario, as long as the native population is reduced by the presence of the exotic predator.

**Table 3:** Existence and stability conditions of the equilibria of the model (2). When $pdK=q$ the equilibria $E_{X}$ and $E_{XY}$ are equals.

Equilibrium	Existence condition	Stability condition
$E_0=(0,0,0)$	Always	Always unstable
$E_X=\left( K,0,0\right)$	Always	Always unstable
$E_{XY}=\left( \frac{q}{dp},\frac{r(pdK-q)}{d^{2}Kp},0\right)$	$pdK-q>0$	$s-\frac{b r(p d K -q)}{d^2 K p}<0$
$$E_{XZ}=\left( \frac{K(r-\alpha dc)}{\alpha ^{2} d^{2}Kn+r},0,\frac{r(c+n\alpha dK)}{\alpha ^{2}d^{2}Kn+r}\right)$$	$$r-\alpha dc>0$$	$$\alpha d^2 K (c p + \alpha n q)$$$$+\left[ \beta b (c + \alpha d K n) - p d K + q\right] r>0$$
$E_Z=(0,0,c)$	Always	$r<\alpha cd$
$E_{s}=\left( X_s,\frac{s(q-pdX_s+\beta b(c+n\alpha dX_s)}{\beta b^{2} (c+n \alpha dX_s)},\frac{pdX_s-q}{\beta b}\right)$	$\beta b(c+n \alpha dX_s)>pdX_s-q>0$	See Stability conditions in Supplementary Material

These regions are subject to change as *b* (competition coefficient) and *d* (predation rate) change. If the parameter *b* increases, region *IV* disappears, meaning that scenario *IV* becomes impossible. Scenario *I* is possible in the whole $(\alpha ,\beta )$-plane when $b>0.01$ (see Fig. 3a–c). This outcome is a consequence of the stability condition of $E_{XY}$ given by $-b r(pd K-q)/\{d^2 K p\}+s<0$, which depends inversely on *b*. This means that the exotic population cannot establish if its intrinsic growth rate *s* is less than its mortality rate due to interspecific competition $by_s$, where $y_s=r(p d K -q)/{d^2 K p}$ represents the abundance reached by the native competitor.

To explore invasion success scenarios, we choose an appropriate value of *b* that includes region *IV*. As the parameter *d* increases, regions *IV* and *II* decrease and region *III* increases (see Fig. 4a–c). Note that region *I* does not appear as *d* increases. This result is a consequence of the stability condition of $E_{XY}$ given by $-b r(p d K -q)/\{d^2 K p\}+s<0$, which remains as *d* increases. Regions *I* and *III* are separated by $\alpha = r/{cd}$, indicated by the vertical line in Figs. 3 and 4. This line represents the threshold predation rate required to maintain the prey population in its presence. This threshold decreases as *d* increases, as shown in Fig. 4a–c.

### Propagule size dependence

**Figure 6:**
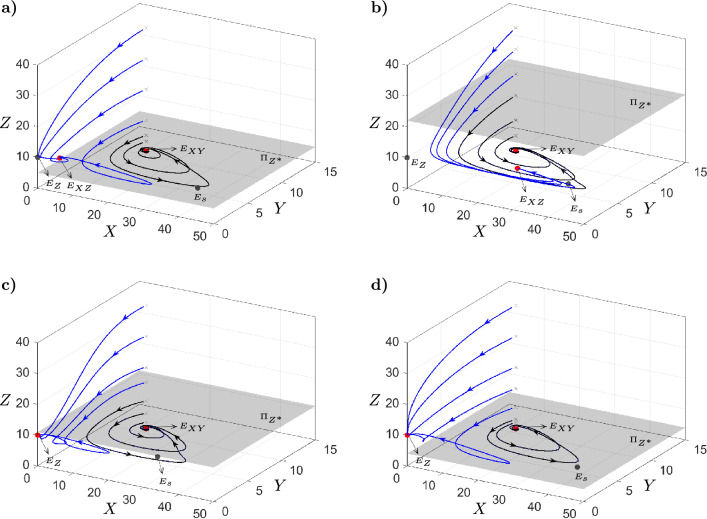
Phase portraits of model (2). In panels (**a**) and (**b**) the blue trajectories converge to the equilibrium point $E_{XZ}$, while panels (**c**) and (**d**) converge to the equilibrium point $E_{Z}$. In panels (**a**–**d**) the black trajectories converge to the equilibrium point $E_{XY}$. The initial conditions of the trajectories are the form of $(X_s,Y_s,Z_0)$, where $X_s$ and $Y_s$ represent the population densities when the native system is at equilibrium. The success of the invasion depends on the initial propagule size $Z_0$. If $Z_0\ge Z^{*}$, the exotic predator becomes established. Conversely, if $Z_0< Z^{*}$, the exotic population cannot grow. Fixed $r=1.2$, $s=0.2$ $K=100$, $c=10$, $n=2$, $p=0.05$, $q=0.05$, $b=0.04$, $d=0.1$ and in panel (**a**) $\alpha =1$ and $\beta =1$, panel (**b**) $\alpha =0.6$ and $\beta =0.5$, panel (**c**) $\alpha =1.2$ and $\beta =0.5$, panel (**d**) $\alpha =1.2$ and $\beta =1.5$. In all panels, under the mentioned parameter values, the initial conditions for the native prey and predator populations are $X_{s}=10$ and $Y_{s}=10.8$, respectively.

**Figure 7:**
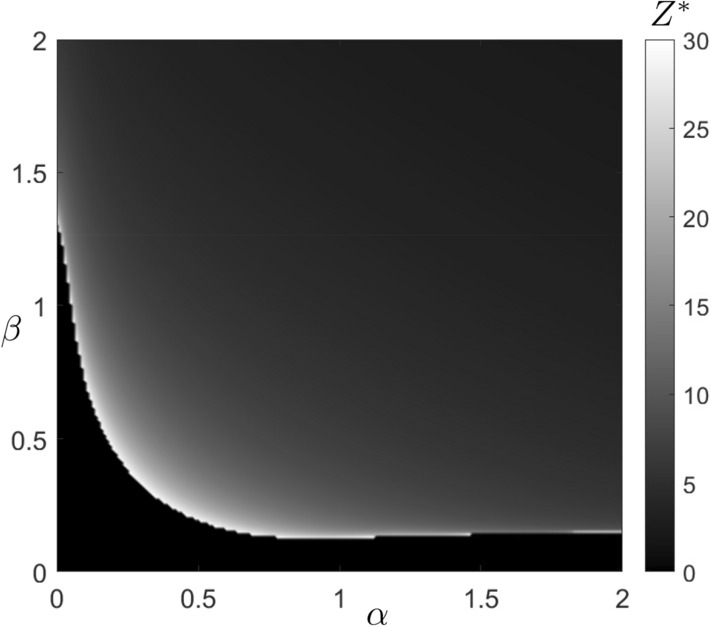
Values of the minimum propagule size, denoted as $Z^{*}$, required for the establishment of the exotic predator are presented on a grayscale in the parameter space $\left( \alpha ,\beta \right)$. For values of $\alpha$ and $\beta$ within the black region, $Z^{*}=0$, indicating that the exotic predator does not establish itself. Fixed $r=1.2$, $s=0.2$ $K=100$, $c=10$, $n=2$, $p=0.05$, $q=0.05$, $b=0.04$ and $d=0.1$. For the mentioned parameter values, the initial conditions for the native prey and predator populations are $X_{s}=10$ and $Y_{s}=10.8$, respectively.

There are regions in the $(\alpha ,\beta )$-plane where the equilibrium points $E_{XY}$ and $E_{XZ}$ (or $E_{Z}$) are stable at the same time. This suggests that the resulting scenario depends on the initial condition $(X_0,Y_0,Z_0)$. Since the exotic predator enters at the native system in equilibrium, the initial population of native prey and predator are given by $X_s$ and $Y_s$, respectively. Therefore, the invasion success depends on the propagule size $Z_0$, which refers to the initially introduced exotic population. Figure 6 shows that for values of $Z_0$ over or above the grey plane, a successful invasion scenario is obtained. Specifically, in panels (a) and (b) the equilibrium $E_{XZ}$ is stable, corresponding to scenario *II*, and in panels (c) and (d) the equilibrium $E_{Z}$ is stable, corresponding to scenario *III*. In addition, the grey plane moves vertically depending on the values of $\alpha$ and $\beta$. For instance, in panel (a) the grey plane is lower compared to the grey plane in panel (b). Defining $Z^{*}$ as the minimum propagule size necessary for the establishment of the exotic predator, Fig. 7 shows how $Z^{*}$ change in the $(\alpha ,\beta )$-plane.

### Persistence of the two native species in the presence of the exotic predator

The positive equilibrium point $E_{s}=(X_s,Y_s,Z_s)$ represents the coexistence of the native species with the exotic predator. This equilibrium is obtained by setting the model equations to zero and solving for the variables (see Positive equilibrium point in Supplementary Material for more details). In this way, the coordinates $Y_{s}$ and $Z_{s}$ depend on $X_{s}$, where $X_{s}$ is a root of the polynomial function

$$\begin{aligned} P(\theta )=A_{0}+A_{1}\theta +A_2\theta ^{2},\quad 0<\theta <K, \end{aligned}$$

with $A_{0}=bcK(\alpha dq+\beta br)-dKs(\beta bc+q)$, $A_{1}=\alpha ^2 b d^2 K n q+a b d K (\beta bn r-d (\beta n s+c p))-\beta b^2 c r+d^2 K p s$ and $A_{2}=-\alpha bnd(\alpha d^2Kp+\beta br)<0$. The number of roots of the polynomial depends on the signs of its coefficients and discriminant. Also, $X_{s}$ must satisfy $0<X_{s}<K$ to be the first coordinate of an equilibrium point. Due to, the parabola $P(\theta )$ is concave downward, then $P(K)<0$ is obtained, which is equivalent to $A_{4}=\alpha b (c + \alpha d K n) (q-d K p) - (\beta b (c + \alpha d K n) - d K p + q) s<0$. Therefore, we state the following proposition.

#### Proposition 2

**Figure 8:**
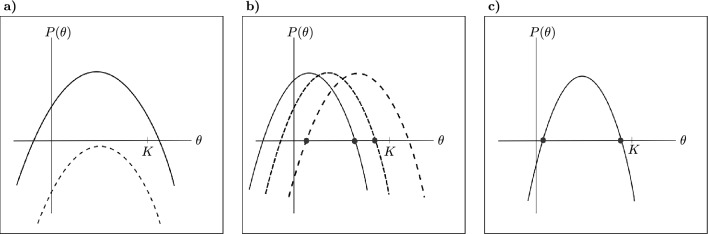
Different plots of the parabola $P(\theta )$. In panel (**a**) there is no positive equilibrium point. In panel (**b**) there is one positive equilibrium point. In panel (**c**) there are two positive equilibrium points. The positive equilibrium of interest is one of greater magnitude because it satisfies the condition $P'(X_s)<0$.

The system (2) can have:

(i) None positive equilibrium point if $A_{1}^{2}-4A_{0}A_{2}<0$ or $A_{0}>0$ and $A_{4}>0$ (see Fig. 8a).
(ii) One positive equilibrium point if $A_{0}>0$ and $A_{4}<0$ or $A_{0}=0$ and $-A_{1}<KA_{2}$ or $A_{0}<0$, $A_{1}^{2}-4A_{0}A_{2}>0$, $A_{1}>0$ and $A_{4}>0$ (see Fig. 8b).
(iii) Two positive equilibrium point if $A_{0}<0$, $A_{1}^{2}-4A_{0}A_{2}>0$, $A_{1}>0$ and $A_{4}<0$ (see Fig. 8c).

#### Proof Proposition 2

Considering the signs of $A_1$, $A_2$, $A_3$, and $A_4$ it obtains conditions for i), ii) and iii).

The equilibrium $E_{s}$ must be stable to ensure the coexistence of native species with the exotic predator over time. A necessary, but not sufficient, stability condition is $P'(X_s)<0$. It follows from Proposition 2, that if there are two positive equilibria, only one is significant. The equilibrium with the smaller value is discarded due to its instability with respect to the $X_s$ component, while the equilibrium with the greater magnitude could potentially lead to asymptotic local stability. Additional stability conditions are given in the following proposition (the proof of Proposition 3 is in Supplementary Material):

#### Proposition 3

The positive equilibrium point $E_{s}=(X_{s},Y_{s},Z_{s})$ is locally asymptotically stable if $P_{1}<0$ and $P_{2}>0$, where $P_1$ and $P_2$ are parameters expression defined latter in Supplementary Material.

### Effects on native population densities

As mentioned above, scenario *IV* shows the persistence of both native species in the presence of the exotic predator. Although the native populations are maintained over time in the presence of the exotic species, their population size may either increase, decrease or remain unchanged. It is therefore, necessary to evaluate the impact of the exotic predator on the density of the native populations. To accomplish this, a measure is introduced that facilitates the comparison of the population size of native prey and native predators in the presence and absence of the exotic predator.

Let *X* and *x* be the size of the native prey population when the exotic predator is present and absent, respectively. Similarly, let *Y* and *y* be the size of the native predator population when the exotic predator is present and absent, respectively. Following the idea of trophic cascade intensity implemented in^57^, it is defined as:

$$\begin{aligned} R_{1}= \frac{X}{x} \quad \text{ and } \quad R_{2}= \frac{Y}{y}. \end{aligned}$$

The values of $R_{1}$ and $R_{2}$ allow us to evaluate the impact of the presence of exotic predator on native population densities. If $R_{1}$ is less than one, it indicates a decrease in the population of native prey, and if $R_{2}$ is less than one, it implies a decrease in the population of native predators. Conversely, if $R_{1}$ is greater than one, it indicates an increase in the population of native prey, and if $R_{2}$ is greater than one, it implies an increase in the population of native predators. The impact on native population densities will depend on predation pressure and the competition intensity of the exotic predator.

The variability of $R_1$ and $R_2$ in the $(\alpha , \beta )$-plane is illustrated to determine how the interplay between predation and competition imposed by the exotic predator affects the native population densities. The numerical simulations consider parameter values derived from bibliographic sources, corresponding to a particular example, the American mink (*Mustela vison*, see Table 4). This exotic species has been introduced into Europe and South America, where it is responsible for declines in native preys including birds^52^ and mammals^53^. This invasive species has also had serious negative impacts on native competitors such as *Lutra lutra* and *Lontra provocax* in Europe and South America, respectively^36,37^.

**Table 4:** Parameters values to the simulation of the model (2) based on America vison (*Mustela vison*) life history.

Param.	Ecological meaning	Values	Unit	Ref.
*r*	Intrinsic growth rate of the native prey	1.2	$year^{-1}$	^58^
*s*	Intrinsic growth rate of the exotic predator	0.2	$year^{-1}$	^58^
*K*	Carrying capacity of native prey	$\left[ 100,200\right]$	*indiv*.	
*d*	Consuming rate per capita of the native predator	$\left[ 0.1,0.3\right]$	$indiv.^{-1} year^{-1}$	^37^
*n*	Enlarger of carrying capacity	3	$indiv.\cdot year$	^59^
*c*	Carrying capacity minimum of exotic predator guaranteed by the alternative food	0.05	*indiv*.	^59^
*b*	Competition coefficient	$\left[ 0.001,0.01\right]$	$indiv.^{-1} year^{-1}$	^36,37^
*p*	Conversion coefficient: food intake to new native predator	$\left[ 0.025,0.05\right]$	unitless	^37^
*q*	Mortality rate of the native predator	$\left[ 0.05,0.1\right]$	$year^{-1}$	^60^
$\alpha$	Constant of proportionality between predation rates	variable	unitless	
$\beta$	Constant of proportionality between competition coefficients	variable	unitless	

**Figure 9:**
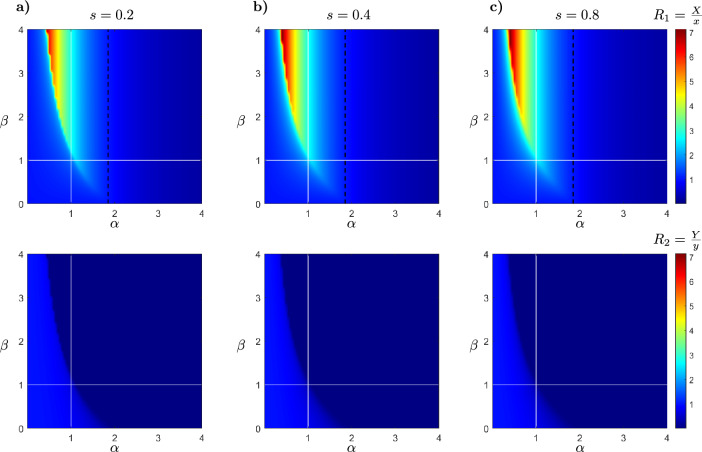
Values of $R_{1}$ and $R_{2}$ presented to color scale for the combination of parameters $\alpha$ and $\beta$. The dotted line represents $R_1=1$ and the white vertical and horizontal lines indicate $\alpha =1$ and $\beta =1$, respectively. In panel (**a**) $s=0.2$, in panel (**b**) $s=0.4$, in panel (**c**) $s=0.8$ and fixed $r=1.2$, $K=100$, $c=0.05$, $n=3$, $p=0.05$, $q=0.05$, $d=0.1$ and $b=0.01$. The initial conditions for native prey, native predator and exotic predator populations are $X_{0}=15$, $Y_{0}=10$ and $Z_{0}=2$, respectively.

Figure 9 illustrates the variability of $R_1$ and $R_2$ within the $(\alpha , \beta )$-plane, represented by a color scale. Considering the presence of the exotic predator, this variability is presented for different values of its intrinsic growth rate, denoted as $s=0.2$, $s=0.4$, and $s=0.8$. Notably, as the value of *s* increases, the red region expands, indicating the enlargement of the domain where the size of the native prey population increases is increased in the presence of the exotic predator. In the top panels, the dotted line represents $R_1=1$, meaning that for $(\alpha , \beta )$ values on the left side of this line, $R_{1}>1$, while on the right side, $R_{1}<1$. In the lower panels, $R_{2}<1$ across the entire $(\alpha , \beta )$-plane. The vertical white line indicates when $\alpha =1$, and the horizontal white line indicates when $\beta =1$. These lines establish a relationship between the values of $R_1$ and $R_2$ with the predation pressure and competition intensity imposed by the exotic predator. Specifically, in scenarios where $\alpha <1$ and $\beta >1$, the prey population size increases in the presence of the exotic predator. This implies that the exotic predator consumes less prey and exhibits strong competition relative to the native predator. Table 5 is constructed to illustrate the impacts on native populations resulting from predation and competition by the exotic predator.

**Table 5:** Different situations of predation and competition between exotic and native predators, and their impact on native populations. Solid arrows represent the strength of predation, indicating the flow of energy from native prey to predators. Dashed arrows represent competition between predators. The thickness of the arrows corresponds to the intensity of the interaction.

Interactions diagram	Consequences on the native populations
	The prey population grows moderately, while the native predator population declines but not becomes extinct (Fig. 10, blue line).
	The prey population grows substantially, depending on the interplay between competition and predation. The native predator population either declines or even becomes extinct. As the competitive abilities of the exotic predator increase, its predation rate must decrease to ensure the survival of the native predator (Fig. 10, green line).
	The prey population grows moderately, while the native predator population either declines or becomes extinct when the predation rate of the exotic predator increases (Fig. 10, greenish-blue line).
	The prey population grows moderately, while the native predator population becomes extinct (Fig. 10, yellow line).
	The prey population decreases and the native predator population becomes extinct, regardless of the competitive abilities of the exotic predator (Fig. 10, magenta line).
	The native prey and predator population become extinct, regardless of the competitive abilities of the exotic predator (Fig. 10, red line).

**Figure 10:**
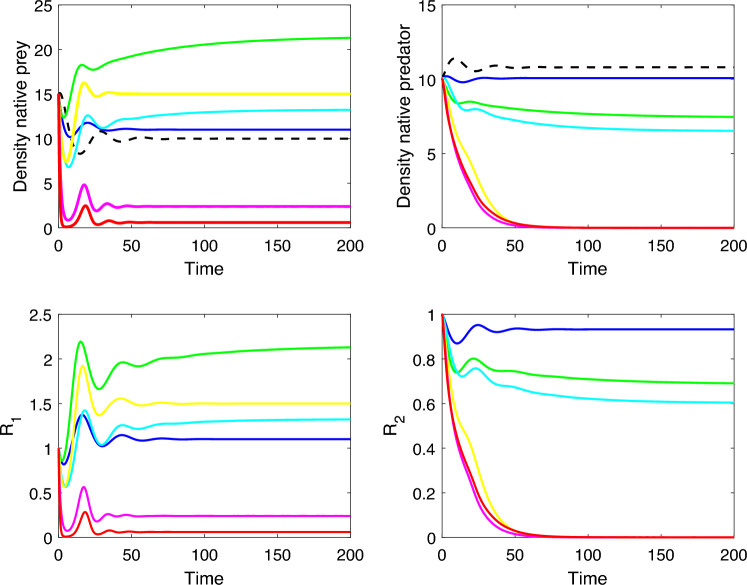
The variation in the native population with and without the presence of the exotic predator. The dotted line represents the native population when the exotic predator is absent, while the colored lines represent the native population when the exotic predator is present. Blue line $\alpha =0.6$ and $\beta =0.5$, green line $\alpha =0.7$ and $\beta =2$, greenish-blue line $\alpha =1.2$ and $\beta =0.5$, yellow line $\alpha =1.5$ and $\beta =2$, magenta line $\alpha =4$ and $\beta =2$ and red line $\alpha =8$ and $\beta =2$, and fixed $s=0.2$, $r=1.2$, $K=100$, $c=0.05$, $n=3$, $p=0.05$, $q=0.05$, $d=0.1$ and $b=0.01$. The initial conditions for native prey, native predator and exotic predator populations are $X_{0}=15$, $Y_{0}=10$ and $Z_{0}=2$, respectively.

## Discussion

This research focused on the construction and study of a mathematical model to determine the effects of predation and competition on invasion success in a native predator-prey system. The exotic species is a generalist predator that consumes the native prey and competes with the native predator. In order to make a comparison between native and exotic predator, predation rates and competition coefficients of both predators were assumed to be proportional. A direct qualitative analysis of the model was conducted to explicitly maintain the parameter *d*. Both interactions and the interplay between them were shown to play a key role in the success of the invasion.

The model describes four possible scenarios: *I* the exotic population is unable to thrive, *II* the exotic population grows, leading to the decline and possible extinction of the native predator, *III* the exotic population grows and the native prey declines to extinction. Consequently, the native predator also declines to extinction, and *IV* the exotic population increases, leading the system to reach a new equilibrium with all three species. The conditions that determine the occurrence of each scenario were established by considering the predation pressure and the intensity of competition imposed by the exotic predator.

Scenarios *II* and *III* correspond to invasion success scenarios because one or both native species become extinct in the presence of the exotic predator. Scenario *IV* can also represent an invasion success scenario despite the persistence of both native species^25,61^. To assess the impact of the exotic predator on the native population density a measure inspired by the trophic cascade effect was defined to determine whether the native population increases or declines in its presence^57^. The native prey population might increase or decrease while the native predator population decreases or disappears in the presence of the exotic predator. This is consistent with the fact that the presence of exotic species may benefit some natives but disadvantage others^62^.

In the proposed model, the population of native predators decreases in the presence of the exotic predator. This outcome may arise from the tendency of generalist exotic predators to deplete resources to levels lower than those reached by native predators. This situation can lead to increased competition for resources, and in some cases, competitive exclusion of native predators^63,64^. For example, the invasion of mink has led to a decline in native polecat numbers^65^ and rainbow and brown trout have led to the displacement of native charr species^66^. However, if the predation rate of the native predator significantly exceeds that of the exotic predator, the native predator may experience population a modest decline, even if it is a weaker competitor.

When the predation rates of both predators are equal, the native predator must have a similar or higher level of competitiveness than the exotic predator to maintain its population. This supports the idea that competitive superiority can influence the success of a biological invasion^67^. However, if the predation rate of the exotic predator exceeds that of the native predator, the native predator may experience population decline or extinction, even though it has superior competitive abilities relative to the exotic predator^68^. Indeed, when the native prey lacks effective anti-predator mechanisms against the exotic predator, it results in a higher exotic predation rate relative to the native predation rate^31,69,70^. Consequently, there is a significant chance of successful invasion.

The native prey population could increase, decrease, or become extinct, depending on a threshold for the exotic predator predation rate^31^. This threshold is defined as the ratio between the intrinsic growth of the prey *r* and the minimum carrying capacity of the exotic predator guaranteed by the alternative food *c*. Moreover, the threshold decreases as the predation rate of the native predator *d* increases. If the predation rate of the exotic predator is above this threshold, then the native system is completely extinct, even if the exotic predator is a weak competitor, with respect to the native predator extinct^71^. Conversely, when the exotic predator predation rate is below this threshold then the prey population could experience modest or substantial growth, and the native predator population could either decline or even become extinct^72^.

The presence of the exotic predator could benefit the prey population^73^. The growth potential of the native prey is determined by the interplay between predation and competition. Specifically, if the exotic predator has lower prey consumption and outperforms the native predator in competitive interactions^74^. Reduced predation pressure from the exotic predator may result in higher survival rates and increased reproduction among the native prey, which leads to population growth^71^. The exotic predator may also exert stronger competitive intensity on the native predator, reducing its population size. This reduction in the abundance of the native predator could create more favorable conditions for the native prey to increase its population size^75^. Therefore, considering these circumstances, the maximum growth of the prey population increases as the intrinsic growth rate of the exotic predator increases.

The establishment of the exotic species depends on the initial propagule size^9,76^, which, in turn, is influenced by biotic resistance^21,22^. From the results, if the exotic predator exerts high predation pressure and intense competition on the native system, then the required initial propagule size for its establishment is smaller than if the predation pressure and competition intensity of the exotic predator were low. Indeed, as the size of the exotic predator’s propagule increases, its predation capacity may intensify, leading to greater competition with native predators for limited resources. This competition could exert additional pressure on native predator populations, subsequently affecting the native prey^11,20^.

The results of this study confirm the fact that invasive species have a negative impact on native species^3,4,6^. There is a high probability of successful invasion by a generalist exotic predator that both preys on and competes with the native prey and predator, respectively^33,34,77^. Although the native prey may benefit from the presence of the exotic when the predation rate of the exotic predator is below the threshold, the negative impact on the native predator is inevitable. Specifically, the American mink (*Mustela vison*) represents a significant threat to native species due to its higher predation rate compared to native predators, which may lead to the extinction of native prey and consequently negative impact on native predators^34,37^.

The mathematical model studied considers a Holling Type-I functional response, which is sufficient to address our research question. However, the choice of the functional response may vary depending on consumption patterns in prey-native predator, prey-exotic predator interactions, and even by interaction native predator-exotic predators^78–80^. The choice of a specific functional response would contribute to a more realistic system description^32,81^. In the context of biological invasion, a Holling Type-II functional response is observed in native and exotic predators, involving a handling time measure^32,82–84^. Alternative functional response types exist for exotic predators, aiming to describe better certain behaviors and their effects on prey populations, including prey handling interference^85–87^. Our strategic model provides a simplified foundation for future research to explore the impact and potential of functional responses on the success of biological invasions.

Finally, the study of mathematical models that allow the study of the effect of the interplay between interspecific interactions on the success of an invasion is an optimal strategy to gain a broader understanding of biological invasions. Given that mathematical models prove to be effective tools for studying biological invasions, it is possible to consider the formulation of new models that can incorporate other important components of the biological invasion process, such as the spread stage or phenotypic changes.

### Supplementary Information

Supplementary Information.

## Data Availability

All data generated or analysed during this study are included in this published article and its supplementary information files.

## References

[1] Clavero M, García-Berthou E (2005). Invasive species are a leading cause of animal extinctions. Trends Ecol. Evolut..

[2] Early R (2016). Global threats from invasive alien species in the twenty-first century and national response capacities. Nat. Commun..

[3] Pyšek P (2020). Scientists’ warning on invasive alien species. Biol. Rev..

[4] Roy, H. E., Pauchard, A., Stoett, P. & Truong, T. R. Ipbes summary for policymakers of the thematic assessment report on invasive alien species and their control of the intergovernmental science-policy platform on biodiversity and ecosystem services. Tech. Rep., IPBES secretariat, Bonn, Germany (2023). 10.5281/zenodo.7430692.

[5] Van Kleunen M, Dawson W, Schlaepfer D, Jeschke JM, Fischer M (2010). Are invaders different? a conceptual framework of comparative approaches for assessing determinants of invasiveness. Ecol. Lett..

[6] Simberloff D (2013). Impacts of biological invasions: What’s what and the way forward. Trends Ecol. Evolut..

[7] Strayer DL, Eviner VT, Jeschke JM, Pace ML (2006). Understanding the long-term effects of species invasions. Trends Ecol. Evolut..

[8] Dodet M, Collet C (2012). When should exotic forest plantation tree species be considered as an invasive threat and how should we treat them?. Biol. Invasions.

[9] Lockwood JL, Cassey P, Blackburn T (2005). The role of propagule pressure in explaining species invasions. Trends Ecol. Evolut..

[10] Rahel FJ, Olden JD (2008). Assessing the effects of climate change on aquatic invasive species. Conserv. Biol..

[11] Stringham OC, Lockwood JL (2021). Managing propagule pressure to prevent invasive species establishments: Propagule size, number, and risk-release curve. Ecol. Appl..

[12] Juliano SA, Lounibos LP, Nishimura N, Greene K (2010). Your worst enemy could be your best friend: Predator contributions to invasion resistance and persistence of natives. Oecologia.

[13] Hill AM, Lodge DM (1999). Replacement of resident crayfishes by an exotic crayfish: The roles of competition and predation. Ecol. Appl..

[14] Noonburg EG, Byers JE (2005). More harm than good: When invader vulnerability to predators enhances impact on native species. Ecology.

[15] Ortega YK, McKelvey KS, Six DL (2006). Invasion of an exotic forb impacts reproductive success and site fidelity of a migratory songbird. Oecologia.

[16] Blackburn TM, Cassey P, Lockwood JL (2009). The role of species traits in the establishment success of exotic birds. Glob. Change Biol..

[17] Starling-Windhof A, Massaro M, Briskie JV (2011). Differential effects of exotic predator-control on nest success of native and introduced birds in New Zealand. Biol. Invasions.

[18] Umetsu CA, Evangelista HBA, Thomaz SM (2012). The colonization, regeneration, and growth rates of macrophytes from fragments: A comparison between exotic and native submerged aquatic species. Aquat. Ecol..

[19] Leffler AJ, James JJ, Monaco TA, Sheley RL (2014). A new perspective on trait differences between native and invasive exotic plants. Ecology.

[20] Holle BV, Simberloff D (2005). Ecological resistance to biological invasion overwhelmed by propagule pressure. Ecology.

[21] Kremer LP, Da Rocha RM (2016). The biotic resistance role of fish predation in fouling communities. Biol. Invasions.

[22] Giachetti CB, Battini N, Bortolus A, Tatian M, Schwindt E (2019). Macropredators as shapers of invaded fouling communities in a cold temperate port. J. Exp. Mar. Biol. Ecol..

[23] Dickman CR (1996). Impact of exotic generalist predators on the native fauna of Australia. Wildl. Biol..

[24] Rilov G (2009). Predator-prey interactions of marine invaders. Biol. Invasions Mar. Ecosyst.: Ecol. Manag. Geogr. Perspect..

[25] Anton A (2019). Global ecological impacts of marine exotic species. Nat. Ecol. Evolut..

[26] Schoener TW (1983). Field experiments on interspecific competition. Am. Nat..

[27] Mougi A (2013). Allelopathic adaptation can cause competitive coexistence. Thyroid Res..

[28] Crawley MJ (1986). The population biology of invaders. Philos. Trans. R. Soc. Lond. B, Biol. Sci..

[29] Polo-Cavia N, López P, Martín J (2014). Interference competition between native Iberian turtles and the exotic Trachemys scripta. Basic Appl. Herpetol..

[30] Zaviezo T, Soares AO, Grez AA (2019). Interspecific exploitative competition between Harmonia axyridis and other coccinellids is stronger than intraspecific competition. Biol. Control.

[31] Ehlman SM, Trimmer PC, Sih A (2019). Prey responses to exotic predators: Effects of old risks and new cues. Am. Nat..

[32] Holling CS (1959). Some characteristics of simple types of predation and parasitism. Can. Entomol..

[33] Salo P, Korpimäki E, Banks PB, Nordström M, Dickman CR (2007). Alien predators are more dangerous than native predators to prey populations. Proc. R. Soc. B: Biol. Sci..

[34] Medina FM (2011). A global review of the impacts of invasive cats on island endangered vertebrates. Glob. Change Biol..

[35] Doherty TS, Glen AS, Nimmo DG, Ritchie EG, Dickman CR (2016). Invasive predators and global biodiversity loss. Proc. Natl. Acad. Sci..

[36] Bonesi L, Chanin P, Macdonald DW (2004). Competition between Eurasian otter Lutra lutra and American mink Mustela vison probed by niche shift. Oikos.

[37] Schuettler E, Carcamo J, Rozzi R (2008). Diet of the American mink Mustela vison and its potential impact on the native fauna of Navarino Island, Cape Horn Biosphere Reserve. Chile. Revista Chilena de Historia Natural.

[38] Pintor LM, Sih A, Bauer ML (2008). Differences in aggression, activity and boldness between native and introduced populations of an invasive crayfish. Oikos.

[39] Taggar AK, McGrath E, Despland E (2021). Competition between a native and introduced pollinator in unmanaged urban meadows. Biol. Invasions.

[40] Simberloff D, Von Holle B (1999). Positive interactions of nonindigenous species: Invasional meltdown?. Biol. Invasions.

[41] Kumschick S (2015). Comparing impacts of alien plants and animals in Europe using a standard scoring system. J. Appl. Ecol..

[42] Lowry E (2013). Biological invasions: A field synopsis, systematic review, and database of the literature. Ecol. Evol..

[43] Kot M (2001). Elements of Mathematical Ecology.

[44] Lewis MA, Petrovskii SV, Potts JR (2016). The Mathematics Behind Biological Invasions.

[45] Misra O, Kushwah P, Sikarwar CS (2013). Effect of resource based exotic goose species on native plant species competing with exotic grass: A model. Proc. Natl. Acad. Sci., India, Sect. A.

[46] Tonnang HE, Nedorezov LV, Ochanda H, Owino J, Löhr B (2009). Assessing the impact of biological control of Plutella xylostella through the application of Lotka–Volterra model. Ecol. Model..

[47] Jones H, White A, Lurz P, Shuttleworth C (2017). Mathematical models for invasive species management: Grey squirrel control on Anglesey. Ecol. Model..

[48] Manna K, Banerjee M (2018). Stationary, non-stationary and invasive patterns for a prey-predator system with additive Allee effect in prey growth. Ecol. Complex..

[49] Gutierrez JB, Teem JL (2006). A model describing the effect of sex-reversed yy fish in an established wild population: The use of a Trojan y chromosome to cause extinction of an introduced exotic species. J. Theor. Biol..

[50] Inoue NK (2022). Quantitative evaluation of the effects of bycatch on native species using mathematical models. Ecol. Model..

[51] Shigesada N, Kawasaki K (1997). Biological Invasions: Theory and Practice.

[52] Craik C (1997). Long-term effects of North American mink Mustela vison on seabirds in western Scotland. Bird Study.

[53] Barreto GR, Rushton SP, Strachan R, Macdonald DW (1998). The role of habitat and mink predation in determining the status and distribution of water voles in England. Anim. Conserv. Forum.

[54] Lotka AJ (1925). Elements of Physical Biology.

[55] Volterra V (1928). Variations and fluctuations of the number of individuals in animal species living together. ICES J. Mar. Sci..

[56] Berryman AA (1992). The orgins and evolution of predator-prey theory. Ecology.

[57] Campillay-Llanos W, Córdova-Lepe FD, Moreno-Gómez FN (2022). Coexistence, energy, and trophic cascade in a three-level food chain integrating body sizes. Front. Ecol. Evol..

[58] Tanner JT (1975). The stability and the intrinsic growth rates of prey and predator populations. Ecology.

[59] Anderson CB (2006). Exotic vertebrate fauna in the remote and pristine sub-Antarctic Cape Horn Archipelago. Chile. Biodivers. Conserv..

[60] Caudera E, Viale S, Bertolino S, Cerri J, Venturino E (2021). A mathematical model supporting a hyperpredation effect in the apparent competition between invasive eastern cottontail and native European hare. Bull. Math. Biol..

[61] Sanders NJ, Gotelli NJ, Heller NE, Gordon DM (2003). Community disassembly by an invasive species. Proc. Natl. Acad. Sci..

[62] Goodenough AE (2010). Are the ecological impacts of alien species misrepresented? A review of the “native good, alien bad” philosophy. Commun. Ecol..

[63] Crowder DW, Snyder WE (2010). Eating their way to the top? mechanisms underlying the success of invasive insect generalist predators. Biol. Invasions.

[64] Sanches FHC (2012). Aggressiveness overcomes body-size effects in fights staged between invasive and native fish species with overlapping niches. PLoS ONE.

[65] Brzeziński M, Zarzycka A, Diserens TA, Zalewski A (2022). Correction to: Does the American mink displace the European polecat? A need for more research on interspecific competition between invasive and native species. Eur. J. Wildl. Res..

[66] Hasegawa K (2020). Invasions of rainbow trout and brown trout in Japan: A comparison of invasiveness and impact on native species. Ecol. Freshw. Fish.

[67] Byers JE (2000). Competition between two estuarine snails: Implications for invasions of exotic species. Ecology.

[68] Whitfield PE (2007). Abundance estimates of the Indo-Pacific lionfish Pterois volitans/miles complex in the western North Atlantic. Biol. Invasions.

[69] Carthey AJ, Bucknall MP, Wierucka K, Banks PB (2017). Novel predators emit novel cues: A mechanism for prey naivety towards alien predators. Sci. Rep..

[70] Anton A, Geraldi NR, Ricciardi A, Dick JT (2020). Global determinants of prey naiveté to exotic predators. Proc. R. Soc. B.

[71] Sinclair ARE (1998). Predicting effects of predation on conservation of endangered prey. Conserv. Biol..

[72] Villanueva MCS, Isumbisho M, Kaningini B, Moreau J, Micha J-C (2008). Modeling trophic interactions in Lake Kivu: What roles do exotics play?. Ecol. Model..

[73] Scavia D, Fahnenstiel GL, Evans MS, Jude DJ, Lehman JT (1986). Influence of salmonine predation and weather on long-term water quality trends in Lake Michigan. Can. J. Fish. Aquat. Sci..

[74] Ritchie EG, Johnson CN (2009). Predator interactions, mesopredator release and biodiversity conservation. Ecol. Lett..

[75] Albins MA (2013). Effects of invasive Pacific red lionfish Pterois volitans versus a native predator on Bahamian coral-reef fish communities. Biol. Invasions.

[76] Forsyth DM, Duncan RP (2001). Propagule size and the relative success of exotic ungulate and bird introductions to New Zealand. Am. Nat..

[77] Raymond WW, Albins MA, Pusack TJ (2015). Competitive interactions for shelter between invasive Pacific red lionfish and native Nassau grouper. Environ. Biol. Fishes.

[78] Hooff RC, Bollens SM (2004). Functional response and potential predatory impact of Tortanus dextrilobatus, a carnivorous copepod recently introduced to the San Francisco Estuary. Mar. Ecol. Prog. Ser..

[79] Radford IJ, Dickinson KJ, Lord JM (2007). Functional and performance comparisons of invasive hieracium lepidulum and co-occurring species in New Zealand. Austral Ecol..

[80] Bollache L, Dick JT, Farnsworth KD, Montgomery WI (2008). Comparison of the functional responses of invasive and native amphipods. Biol. Let..

[81] Holling, C. S. The strategy of building models of complex ecological systems. *Syst. Anal. Ecol.* 195–214 (1966).

[82] Courchamp F, Langlais M, Sugihara G (1999). Control of rabbits to protect island birds from cat predation. Biol. Cons..

[83] Křivan V, Eisner J (2006). The effect of the holling type ii functional response on apparent competition. Theor. Popul. Biol..

[84] Pei Y, Zeng G, Chen L (2008). Species extinction and permanence in a prey-predator model with two-type functional responses and impulsive biological control. Nonlinear Dyn..

[85] Crowley PH, Martin EK (1989). Functional responses and interference within and between year classes of a dragonfly population. J. N. Am. Benthol. Soc..

[86] Misra O, Kushwah P, Sikarwar CS (2012). Effect of exotic species on a system of native prey-predator populations: a model. Am. J. Comput. Appl. Math..

[87] Parshad RD, Basheer A, Jana D, Tripathi JP (2017). Do prey handling predators really matter: Subtle effects of a Crowley–Martin functional response. Chaos Solit. Fract..

