# Supplementary material for: Exploring the effects of competition and predation on the success of biological invasion through mathematical modeling
Source: Sci Rep. 2024 Feb 22;14:4416. doi: 10.1038/s41598-024-53344-1 (PMC10883959; doi:10.1038/s41598-024-53344-1)
Supplement: Supplementary file 1 — Supplementary Information. [file 41598_2024_53344_MOESM1_ESM.pdf]

Supplementary Material

Exploring the effects of competition and predation on the success of biological invasion through mathematical modeling

Stability conditions

Lemma 1. *The equilibrium $E_0 = (0,0,0)$ is a hyperbolic saddle for all positive parameter values.*

Proof Lemma 1. *Evaluating the Jacobian matrix of the system (2) in the equilibrium E_0 the eigenvalues are $\lambda_1 = -q$, $\lambda_2 = r$ and $\lambda_3 = s$. Therefore, the equilibrium E_0 is a hyperbolic saddle point.* □

Lemma 2. *The equilibrium $E_X = (K,0,0)$ is a hyperbolic saddle for all parameter values.*

Proof Lemma 2. *Evaluating the Jacobian matrix of the system (2) in the equilibrium E_X the eigenvalues are $\lambda_1 = dKp - q$, $\lambda_2 = -r$ y $\lambda_3 = s$. Therefore, the equilibrium E_X is a hyperbolic saddle.* □

Lemma 3. *The equilibrium $E_Z = (0,0,c)$ is a:*

- *stable node if $r - \alpha cd < 0$,*
- *no hyperbolic if $r - \alpha cd = 0$, or*
- *hyperbolic saddle if $r - \alpha cd > 0$.*

Remark 1. *The first statement indicates that when the native predator is absent and the prey density is low, if the intrinsic growth rate of prey r is less than the consumption $(\alpha d)c$ of the exotic predator at its minimum carrying capacity c, then the prey population cannot increase. In fact, $X'/X = \alpha dc(\mathscr{R} - 1)$ holds, where $\mathscr{R} = r/(\alpha dc)$, and the signs correspond to the scenarios outlined in Lemma 3. If $X < [q + \beta bc]/[pd]$, the same situation occurs when considering small Y.*

Proof Lemma 3. *Evaluating the Jacobian matrix of the system (2) in the equilibrium E_Z the eigenvalues are $\lambda_1 = -\beta bc - q$, $\lambda_2 = r - \alpha dc$ y $\lambda_3 = -s$. Therefore, the stability of E_Z depends on the expression sign $r - \alpha cd$.* □

Lemma 4. *The equilibrium $E_{XY} = \left(\frac{q}{dp}, \frac{r(dKp-q)}{d^2Kp}, 0\right)$ is a:*

- *stable focus if $\mu < 0$ and $\rho < 0$,*
- *stable node if $\mu < 0$ and $\rho > 0$,*
- *unestable focus if $\mu > 0$ and $\rho < 0$, or*
- *unestable node if $\mu > 0$ and $\rho > 0$,*

with $\mu = s - \frac{b(dKp-q)}{d^2Kp}$, $\rho = qr - 4dKp(dKp - q)$.

Proof Lemma 4. *Evaluating the Jacobian matrix of the system (2) in the equilibrium E_{XY} the eigenvalues are:*

$$\lambda_{1,2} = -\frac{qr \pm \sqrt{qr\,[qr - 4dKp(dKp-q)]}}{2dKp} = -\frac{qr \pm \sqrt{qr\rho}}{2dKp},$$

$$\lambda_3 = s - \frac{b(dKp-q)}{d^2Kp} = \mu.$$

Note that $Re(\lambda_1)$ y $Re(\lambda_2)$ is always negative. Then, the equilibrium point E_{XY} is stable if $\lambda_3 < 0$. Also, λ_1 and λ_2 are real numbers if $\rho = qr - 4dKp(dKp - q) > 0$. □

Remark 2. *Note that*

$$\lambda_{1,2} = -\frac{rx_s}{2K}\left[1 \pm \sqrt{1 - \frac{4qK}{rx_s}\left(\frac{K}{x_s} - 1\right)}\right].$$

If $x_s = K$ ($pdK = q$) then $\lambda_1 = -r$, $\lambda_2 = 0$ and $E_{xy} = E_x$.

Lemma 5. *The equilibrium $E_{XZ} = \left(\frac{K(r-\alpha cd)}{\alpha^2 d^2 Kn + r}, 0, \frac{r(\alpha dKn + c)}{\alpha^2 d^2 Kn + r}\right)$ is a:*

- *stable node if $\phi_1 > 0$ and $\phi_2 > 0$,*
- *stable focus if $\phi_1 < 0$ and $\phi_2 > 0$,*
- *unstable node if $\phi_1 > 0$ and $\phi_2 < 0$, or*
- *unstable focus if $\phi_1 < 0$ and $\phi_2 < 0$,*

with $\phi_1 = \left(\alpha^2 d^2 Kns + r(r - \alpha cd) + rs\right)^2 - 4s(r - \alpha cd)^2$, $\phi_2 = \alpha d^2 K(cp + \alpha nq) + (\beta b(c + \alpha dKn) - dKp + q)r$.

Proof Lemma 5. *Evaluating the Jacobian matrix of the system (2) in the equilibrium E_{XZ} the eigenvalues are:*

$$\lambda_1 = -\frac{\alpha d^2 K(cp + \alpha nq) + (\beta b(c + \alpha dKn) - dKp + q)r}{\alpha^2 d^2 Kn + r} = -\frac{\phi_2}{\alpha^2 d^2 Kn + r},$$

$$\lambda_{2,3} = -\frac{\varphi \pm \sqrt{\varphi^2 - 4s(r - \alpha cd)\left(\alpha^2 d^2 Kn + r\right)^2}}{2\left(\alpha^2 d^2 Kn + r\right)}, \quad \text{with} \quad \varphi = \alpha^2 d^2 Kns + r(r - \alpha cd) + rs > 0.$$

Note that $Re(\lambda_2)$ y $Re(\lambda_3)$ is always negative. Therefore, the equilibrium point E_{XZ} is stable if $\lambda_1 < 0$. Also, λ_2 and λ_3 are real number if $\phi_1 = \varphi^2 - 4s(r - \alpha cd)\left(\alpha^2 d^2 Kn + r\right)^2 > 0$ □

Positive equilibrium point

Considering the equations of the model (2)

$$\frac{dX}{dt} = rX\left(1 - \frac{X}{K}\right) - dXY - \alpha dXZ = 0, \tag{1}$$

$$\frac{dY}{dt} = pdXY - \beta bYZ - qY = 0, \tag{2}$$

$$\frac{dZ}{dt} = sZ\left(1 - \frac{Z}{n\alpha dX + c}\right) - bZY = 0. \tag{3}$$

Solving for Z in (2), it obtain $Z = 0$ and $Z(X) = (pdX - q)/\beta b$. Then, replacing $Z(X)$ and solving for Y in (3), it obtain $Y(X) = \left[s\left(\beta bc + q + \alpha\beta bdnX - dpX\right)\right] / \left[\beta b^2\left(c + \alpha dnX\right)\right]$. Finally, replacing $Z(X)$ and $Y(X)$ in (1), it obtain

$$\frac{dX}{dt} = X\left[-\frac{ds(\beta b(c + \alpha dnX) - dpX + q)}{\beta b^2(\alpha dnX + c)} + \frac{\alpha d(q - dpX)}{\beta b} - \frac{rX}{K} + r\right] = 0, \tag{4}$$

such that if X_s is solution of (4) if only if is a non-zero roots of the quadratic polynomial $P(\theta) = A_0 + A_1\theta + A_2\theta^2$, with $A_0 = bcK(\alpha dq + \beta br) - dKs(\beta bc + q)$, $A_1 = \alpha^2 bd^2 Knq + abdK(\beta bnr - d(\beta ns + cp)) - \beta b^2 cr + d^2 Kps$ and $A_2 = -\alpha bnd(\alpha d^2 Kp + \beta br) < 0$. Thus, $Y_s = Y(X_s)$ and $Z_s = Z(X_s)$ are obtained.

Proof Proposition 3 *The characteristic polinomial of the Jacobian matrix at $E_s = (X_s, Y_s, Z_s)$ is*

$$P(\xi) = \xi^3 + B_2\xi^2 + B_1\xi + B_0,$$

where

$$\begin{aligned}
B_0 &= \frac{-X_sY_sZ_s}{K(\alpha dnX_s+c)^2}\left[\alpha bdnX_s(\alpha dnX_s+2c)(\alpha d^2Kp+\beta br)+\alpha bc^2d^2Kp-\alpha d^2Knqs+\beta b^2c^2r\right.\\
&\quad \left.-cd^2Kps\right],\\
B_1 &= s\left\{\frac{-d^2X_sZ_s\left[\alpha^2nq+\alpha dnpx(\beta-\alpha)+\beta cp\right]}{\beta b(\alpha dnX_s+c)^2}+\frac{K\left[d^2px(\alpha\beta dnX_s+\beta c+pX_s)-2dpqX_s+q^2\right]+rX_s(dpX_s-q)}{\beta bK(\alpha dnX_s+c)}\right.\\
&\quad \left.-dpX_s+q\right\},\\
B_2 &= \frac{sZ_s}{c+dnX_s}+\frac{rX_s}{K}>0, \quad \text{and}\\
B_1B_2-B_0 &= s\left\{\frac{\alpha(\alpha-\beta)d^2nsX_sZ_s^3}{(c+\alpha dnX_s)^3}+\frac{rsX_sZ_s}{K(c+\alpha dnX_s)^2}+\frac{rpd^2X_s^2Z_s}{Kb(c+\alpha dnX_s)}+\frac{r\alpha^2nd^2X_s^2Z_s}{K(c+\alpha dnX_s)^2}-\frac{qsZ_s}{(c+\alpha dnX_s)}-\frac{\alpha d^2pX_sZ_s}{(c+\alpha dnX_s)}\right.\\
&\quad \left.+\frac{dsX_sZ_s(\alpha\beta dn-p)}{(c+\alpha dnX_s)^2}+\frac{d^2prX_s^2}{b}+\frac{r^2X_s^2Z_s}{K^2(c+\alpha dnX_s)}+\frac{\beta bsZ_s}{(c+\alpha dnX_s)}-\alpha d^2pX_sZ_s\right\}.
\end{aligned}$$

Assume

$$\begin{aligned}
P_1 &= \alpha^2bd^2n^2X_s^2\left(\alpha d^2Kp+\beta br\right)+2\alpha bcdnX_s\left(\alpha d^2Kp+\beta br\right)+\alpha bc^2d^2Kp-\alpha d^2Knqs+b^2Bc^2r-cd^2Kps<0,\\
P_2 &= \frac{\alpha(\alpha-\beta)d^2nsX_sZ_s^3}{(c+\alpha dnX_s)^3}+\frac{rsX_sZ_s}{K(c+\alpha dnX_s)^2}+\frac{rpd^2X_s^2Z_s}{Kb(c+\alpha dnX_s)}+\frac{r\alpha^2nd^2X_s^2Z_s}{K(c+\alpha dnX_s)^2}-\frac{qsZ_s}{(c+\alpha dnX_s)}-\frac{\alpha d^2pX_sZ_s}{(c+\alpha dnX_s)}\\
&\quad +\frac{dsX_sZ_s(\alpha\beta dn-p)}{(c+\alpha dnX_s)^2}+\frac{d^2prX_s^2}{b}+\frac{r^2X_s^2Z_s}{K^2(c+\alpha dnX_s)}+\frac{\beta bsZ_s}{(c+\alpha dnX_s)}-\alpha d^2pX_sZ_s>0.
\end{aligned}$$

Then, $B_0 > 0$, $B_1 > 0$ and $B_2B_1 - B_0 > 0$. By Routh–Hurwitz criterion the positive equilibrium point is locally asymptotically stable. □
